# A Biosensor-Based Leaf Punch Assay for Glutamine Correlates to Symbiotic Nitrogen Fixation Measurements in Legumes to Permit Rapid Screening of Rhizobia Inoculants under Controlled Conditions

**DOI:** 10.3389/fpls.2017.01714

**Published:** 2017-10-09

**Authors:** Malinda S. Thilakarathna, Nicholas Moroz, Manish N. Raizada

**Affiliations:** Department of Plant Agriculture, University of Guelph, Guelph, ON, Canada

**Keywords:** legume, symbiotic nitrogen fixation, nodule, rhizobia, biosensor, glutamine

## Abstract

Legumes are protein sources for billions of humans and livestock. These traits are enabled by symbiotic nitrogen fixation (SNF), whereby root nodule-inhabiting rhizobia bacteria convert atmospheric nitrogen (N) into usable N. Unfortunately, SNF rates in legume crops suffer from undiagnosed incompatible/suboptimal interactions between crop varieties and rhizobia strains. There are opportunities to test much large numbers of rhizobia strains if cost/labor-effective diagnostic tests become available which may especially benefit researchers in developing countries. Inside root nodules, fixed N from rhizobia is assimilated into amino acids including glutamine (Gln) for export to shoots as the major fraction (amide-exporting legumes) or as the minor fraction (ureide-exporting legumes). Here, we have developed a new leaf punch based technique to screen rhizobia inoculants for SNF activity following inoculation of both amide exporting and ureide exporting legumes. The assay is based on measuring Gln output using the *GlnLux* biosensor, which consists of *Escherichia coli* cells auxotrophic for Gln and expressing a constitutive *lux* operon. Subsistence farmer varieties of an amide exporter (lentil) and two ureide exporters (cowpea and soybean) were inoculated with different strains of rhizobia under controlled conditions, then extracts of single leaf punches were incubated with *GlnLux* cells, and light-output was measured using a 96-well luminometer. In the absence of external N and under controlled conditions, the results from the leaf punch assay correlated with ^15^N-based measurements, shoot N percentage, and shoot total fixed N in all three crops. The technology is rapid, inexpensive, high-throughput, requires minimum technical expertise and very little tissue, and hence is relatively non-destructive. We compared and contrasted the benefits and limitations of this novel diagnostic assay to methods.

## Introduction

Legumes are critical to human and livestock agricultural systems (Broughton et al., 2003; Leigh, 2004; Foyer et al., 2016), as they fix atmospheric nitrogen (N) into a usable form of N (NH_3_/${\mathrm{NH}}_{4}^{+}$) through symbiotic association with rhizobia bacteria inside underground root nodule organs (Udvardi and Poole, 2013). The enzyme glutamine (Gln) synthetase assimilates ${\mathrm{NH}}_{4}^{+}$ into amino acids (Bernard and Habash, 2009; Betti et al., 2012). Therefore, symbiotic nitrogen fixation (SNF) provides the essential building block for amino acid biosynthesis (Prell and Poole, 2006; Udvardi and Poole, 2013), enabling legumes such as lentil and soybean to be a primary source of high-quality protein for billions of people especially in developing nations, and for livestock especially in wealthier societies (Graham and Vance, 2003). SNF also enables the deposition of organic fertilizer into soil during litter decomposition (Thilakarathna et al., 2015, 2016a), thus reducing the need for synthetic N fertilizers, of which subsistence farmers are primary beneficiaries. Unfortunately, some legume species and varieties show low SNF rates, in part due to incompatible or suboptimal interactions with strains of available soil rhizobia (Thilakarathna and Raizada, 2017). Although improved rhizobia can be introduced, screening a panel of rhizobia strains against local crop varieties/landraces for improved SNF is expensive especially for subsistence farmers in developing nations, in part because of the limitations of current SNF diagnostic methods (Unkovich and Pate, 2000; Howieson and Dilworth, 2016). Symbiotic nitrogen fixation activity is currently measured using different methods including counting the number of differentiated rhizobia (bacteroids) per nodule (Bourcy et al., 2013), N difference assay (Unkovich et al., 2008), ureide assay (Unkovich et al., 2008), acetylene reduction assay (Lodwig et al., 2003; Starker et al., 2006), hydrogen production (Kiers et al., 2003; Cabeza et al., 2015), and ^15^N techniques (Lodwig et al., 2003), of which ^15^N is the most commonly used and considered to be the most accurate (Howieson and Dilworth, 2016). These methods are challenging in terms of time, labor, accuracy, and the need for a non-fixing reference plant, destructive sampling, large amounts of tissue, technical expertise, expensive reagents, and/or equipment (Herridge et al., 2008; Howieson and Dilworth, 2016). Therefore, there is a need for alternative high-throughput SNF diagnostic methods.

**FIGURE 1:**
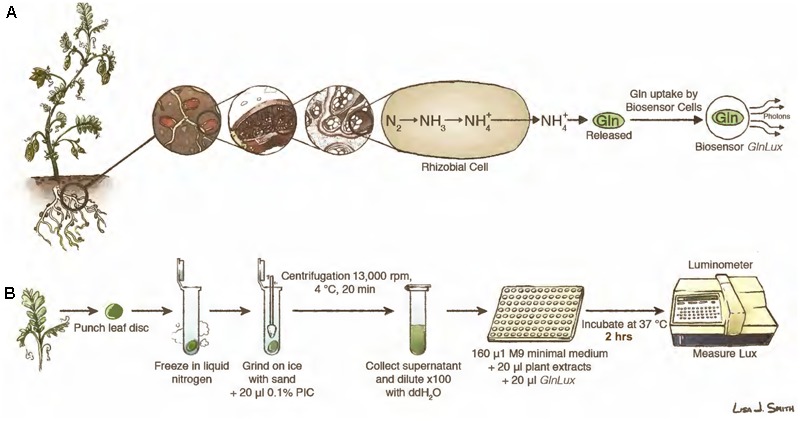
Leaf punch assay to measure symbiotic nitrogen fixation (SNF) in legumes using the *GlnLux* biosensor methodology. **(A)** Principle underlying the leaf punch assay. Legume plants have root nodules that host rhizobia, which following differentiation into bacteroids, convert atmospheric nitrogen (N) into ${\mathrm{NH}}_{4}^{+}$, which is then assimilated into Gln. When Gln is released from plant tissues to the medium, it is taken up by *GlnLux* biosensor cells, causing them to become metabolically active and divide, which proportionally activates a lux operon to release photons that are measured using photon capture devices. **(B)** Overview of the assay using extracts of leaf punches incubated with *GlnLux* biosensor cells in 96-well plates and measured using a luminometer (see section Materials and Methods). Images courtesy of Lisa Smith (University of Guelph) can be re-used under the Creative Commons CC-BY License.

Inside root nodules, fixed N from rhizobia is assimilated into Gln (Prell and Poole, 2006; Tegeder, 2014; **Figure 1A**) for export to shoots as amino acids as the major fraction (amide-exporting legumes) or as the minor fraction (10–20% in ureide-exporting legumes) (Pate et al., 1980; Atkins, 1987). Generally, legumes that originate in temperate regions (e.g., alfalfa, pea, clover) are amide exporters, whereas those that originate in the tropics and subtropics (e.g., soybean, cowpea) predominately export fixed N as ureide compounds (e.g., allantoin) (Unkovich et al., 2008). Whole cell biosensors have been engineered to detect metabolites and emit outputs in response (e.g., fluorescence, luminescence) that can be easily measured, substituting for more expensive analytical chemistry methods such as liquid chromatography mass spectrometry (LC-MS) (Goron and Raizada, 2014). We previously developed a rapid leaf punch-based assay for Gln using a whole cell Gln biosensor called *GlnLux* (Tessaro et al., 2012). *GlnLux* was created by transforming an *Escherichia coli* Gln auxotroph with a constitutive lux reporter (Tessaro et al., 2012). Advantages of an auxotroph compared to expression-based reporter fusions are that there is an absolute requirement for the metabolite (i.e., Gln) and significantly fewer artifacts caused by microbial or plant metabolism or signaling. We showed that *GlnLux* cells exposed to extracts of leaf punches taken across the veins of growing leaves can accurately report N availability in maize (Tessaro et al., 2012; Goron and Raizada, 2016; Goron et al., 2017).

Here, we tested the hypothesis that the *GlnLux* leaf punch assay can be used to infer SNF output for high-throughput screening of legume inoculants under controlled conditions with minimal exogenous N (**Figure 1A**). Extracts of single leaf punches spanning veins of young legume leaves were incubated with *GlnLux* cells in 96-well liquid culture plates, then photon emissions were measured using a plate luminometer (**Figure 1B**). In order to validate the *GlnLux* leaf punch bioassay, two approaches were used: evaluation of different rhizobia strains using both amide and ureide exporting legumes. The crops used were subsistence Nepalese varieties of lentil (*Lens culinaris*) (an amide exporter), a legume of critical importance to South Asia, the Middle East, and East Africa (Sharpe et al., 2013); cowpea (*Vigna unguiculata* L. walp.) (a ureide exporter), a drought-tolerant legume grown in Sub-Saharan Africa, South Asia, and the Caribbean (Ehlers and Hall, 1997); and soybean (*Glycine max*) (a ureide exporter), the world’s most important legume in terms of production (Herridge et al., 2008). The results of *GlnLux* were compared to traditional SNF diagnostic methods including the standard ^15^N dilution technique. Plants were grown with minimal N fertilizer to facilitate N-isotope analysis.

## Materials and Methods

### Plant Materials and *Rhizobium* Strains

For the lentil × rhizobia strain experiment, the crop variety was Nepalese Simal, and there were four *Rhizobium leguminosarum* biovar *viciae* strains (wild type: 3841, VF39, 248; mutant 17-B) (Yost et al., 2006; Vanderlinde et al., 2011). For the cowpea × rhizobia strain experiment, the crop variety was Nepalese Surya and there were two strains of *Bradyrhizobium yuanmingense* (TTC9, TSC10), one strain of *B. japonicum* (DTB4) and one strain of *B. elkanii* (DTC9) (Sarr et al., 2009). For the soybean × rhizobia strain experiment, the crop variety was Nepalese Puja and there were three strains of *B. japonicum* (USDA 110, USDA 123, USDA 510) (Sadowsky et al., 1987; Bhagwat et al., 1991; Kaneko et al., 2002) and two strains of *B. elkanii* (USDA 94, USDA 76) (Kuykendall et al., 1992). Different rhizobia strains for each crop were selected to capture a diversity of nodulation and N fixation efficiency.

### Plant Growth Conditions and Treatments

Seeds of all three crops were surface-sterilized with 70% ethanol for 2 min, 4% sodium hypochlorite for 3 min, and washed with six changes of sterile distilled water. Seeds were pre-germinated on sterilized, wetted filter paper in the dark at 28°C for 2 days, and individual seeds were transferred to germination pouches (17.8 × 16.5 cm, Mega International, Minneapolis, MN, United States) on light shelves containing 50 ml of double distilled water. Each plant was grown in a separate germination pouch. There was only one treatment (one rhizobia strain or non-inoculated control) per pouch, and each germination pouch was considered to be a single replicate. To further reduce cross-contamination, pouches were used that were sealed at the bottom, and nutrient solutions were not shared between treatments. For the lentil and cowpea studies, per treatment, five to six germination pouches were placed in a stand and placed in an open tupperware container. Different rhizobia treatments were not placed within the same container to avoid cross contamination of rhizobia strains. There were three containers per treatment, which were continuously randomized within and across light shelves. For the soybean study, five rhizobia treatments and the non-inoculated control (in individual germination pouches) were randomly allocated within a plastic container. There were eight trays (*n* = 8), which were continuously randomized within and across light shelves. After 1 week of germination, the water was removed and plants were supplied with 50 (lentil) and 100 ml (cowpea and soybean) of one-fourth strength N-free Hoagland’s nutrient solution (pH = 6.6), containing 0.5 mM K^15^NO_3_ (98 atom% ^15^N; 335134-1G; Sigma Aldrich, Oakville, ON, Canada) as starter N. One week later, this nutrient solution was removed and replaced with 50 ml of the one-fourth strength N-free Hoagland’s solution; the nutrient solution was replaced each week. Lentil plants were grown at 23 ± 2°C with supplemental lighting (range: 180–200 μmol m^-2^ s^-1^ at the top of the growth pouch, EcoLux SP65, 40W, F40SP65ECO), maintaining a photoperiod of 16 h/8 h light/dark cycles. Cowpea and soybean plants were grown at 23 ± 2°C with supplemental lighting (range: 250–300 μmol m^-2^ s^-1^ at the top of the growth pouch, EcoLux SP65, 40W, F40SP65ECO), maintaining a photoperiod of 16 h/8 h light/dark cycles. For the rhizobia treatments, strains for lentil (*R. leguminosarum* biovar *viciae* strains wild type: 3841, VF39, 248; mutant 17-B), cowpea (*B. yuanmingense* TTC9, *B. yuanmingense* TSC10, *B. japonicum* DTB4, *B. elkanii* DTC9), and soybean (*B. japonicum* USDA 110, *B. japonicum* USDA 123, *B. japonicum* USDA 510, *B. elkanii* USDA 94, *B. elkanii* USDA 76) were grown in tryptone-yeast (TY) extract and modified arabinose-gluconate medium (MAG) (cowpea and soybean) agar media, respectively, in Petri dishes for 3 days at 30°C. To prepare each inoculum, the agar plates were scraped and cells added to sterilized ddH_2_O, adjusted to a cell density of OD_595_ = 0.1. Plants were inoculated 1 week after plant growth with 1 ml of each inoculum, placed directly on roots. All the three legume species had a negative control, comprised of plants that were not inoculated with rhizobia.

### *GlnLux* Leaf Punch Sampling

Eight healthy representative plants per treatment were sampled from lentil, cowpea, and soybean 4 weeks after inoculation with rhizobia as indicated. Leaf punches were collected from the fully expanded youngest leaf, across the leaflet main vein from lentil (from one leaflet at the tip), cowpea, and soybean using a 3 mm (lentil) and 6.35 mm (cowpea and soybean) hand puncher (235270975; Fiskars Brands Inc., Middleton, WI, United States) and immediately frozen in liquid N. Leaf punches were stored at -80°C.

### Plant Morphometric Analysis

The same eight plants per treatment harvested for *GlnLux* measurements were used. The number of nodules per plant was counted manually. Roots were scanned using an Epson Expression 1640× scanner (Epson Canada Ltd., Markham, ON, Canada), and a detailed root morphological analysis was undertaken using WinRHIZO software (Regent Instruments Inc., Quebec City, QC, Canada) including root volume, total length, surface area, and average diameter. Shoot and root dry weights were measured after drying the plant materials in an oven at 60°C for 3 days.

### Shoot Nitrogen and ^15^N Analysis

The same eight plants per treatment harvested for *GlnLux* measurements were used. The dried shoot samples (see above) were ground using a coffee grinder followed by a Bead Ruptor 12 Homogenizer (OMNI International, Kennesaw, GA, United States) and analyzed for ^15^N and total N% using a mass spectrometer (Costech ECS4010 Elemental Analyzer coupled to a Delta V mass spectrometer, Costech, CA, United States) at the Stable Isotope Facility, University of Saskatchewan, and University of British Columbia, Canada, using a standard protocol (Thilakarathna et al., 2012, 2016b). The %Ndfa of the lentil and soybean was calculated using the following formula according to the isotope dilution technique:

$$\%\;\mathrm{Ndfa}\;=\;\left(1-\frac{\mathrm{atom}\;\%{\;}^{15}N\;{\mathrm{excess}}_{\left(lentil/soybean-inoculated\right)}}{\mathrm{atom}\;{\%}^{15}N\;{\mathrm{excess}}_{\left(lentil/soybean-uninoculated\right)}}\right)\times 100,$$

where atom % ^15^N excess = atom % ^15^N_(lentil/soybean)_ - 0.3663.

The amount of shoot N derived from SNF was calculated based on the shoot N content and %Ndfa (shoot N content × %Ndfa/100).

### Biosensor Strain

Construction of the *GlnLux* biosensor strain was previously reported (Tessaro et al., 2012). Briefly, a Gln-auxotrophic *E. coli* strain (JW3841-1, KanR) was generated by inserting a kanamycin cassette into *GlnA* [glnA732(del)::kan] (Baba et al., 2006). The strain was subsequently transformed with ampicillin-resistant plasmid pT7-lux (Meighen and Szittner, 1992) which contained a constitutive T7 promoter from *Xenorhabdus luminescens* driving expression of the *luxCDABE* operon from *Vibrio fischeri* to create strain *GlnLux*.

### *GlnLux* Bacterial Growth Media

*GlnLux* bacteria were cultured in LB medium, which consisted of 5 g/l NaCl (BP358-212, Fisher Scientific), 5 g/l yeast extract (DF0127179, Fisher Scientific), and 10 g/l tryptone (BP1421-500, Fisher Scientific), with or without 12 g/l Bacto-Agar (BD; DF0140010, Fisher Scientific), pH 7.2. M9 minimal medium consisted of 20 ml/l 20% (w/v) D-(+)-glucose (G5767, Sigma), 100 μl/l 1 M CaCl_2_ (C-79, Fisher Scientific), 2 ml/l 1 M MgSO_4_ (230391, Sigma), and 200 ml/l 5× M9 salts (A-0171, Sigma), pH 7.0. All liquid and solid plate media were supplemented with 50 μg/ml kanamycin monosulfate (K378, PhytoTech, United States) and 100 μg/ml carbenicillin disodium salt (C346, PhytoTech, United States) to select for the disrupted *glnA* chromosome and reporter plasmid, respectively.

### Leaf Punch Luminometer Assays – *GlnLux* Cell Preparation

Cells were prepared as previously described (Tessaro et al., 2012). Briefly, *GlnLux* bacteria were inoculated into 15 ml of LB medium in a 50 ml Falcon tube, and incubated overnight at 37°C with shaking at 250 × *g*. The culture was spun down at 2500 × *g* at 21°C for 10 min and the supernatant was decanted. The culture was then washed 3× in sterile M9 minimal medium with centrifugation as above. Finally, the culture was resuspended in 15 ml sterile M9 minimal medium in a 50 ml Falcon tube, and the *GlnLux* density was adjusted to OD_595_ = 0.025 using sterile M9 minimal medium. The resuspended *GlnLux* culture was incubated at 37°C with shaking at 250 × *g* for 14 h to deplete any endogenous Gln.

### *GlnLux* Leaf Punch Luminometer Measurements of Plant Gln

The procedure was adapted from a previous protocol (Tessaro et al., 2012). Individual frozen leaf punches were ground in a 2 ml conical bottom microcentrifuge tube placed on ice using a micropestle (K7495150000, Kimble Chase, Fisher Scientific) with silica sand in 20 μl of 0.1% final (v/v) protease inhibitor cocktail (PIC) for plant cell extracts (100% stock; #P9599, Sigma). Plant extracts were centrifuged for 20 min at 4°C at 13,000 × *g*, and the supernatant was transferred to a microcentrifuge tube placed on ice. The plant extracts were diluted 100-fold in ddH_2_O. The diluted plant extracts were used for luminometer assays.

For luminometer assays, white opaque 96-well reader plates (#07-200-589, Fisher Scientific) were loaded with 160 μl/well M9 minimal medium followed by 20 μl/well of each plant extract. Finally, 20 μl of 14 h-Gln-depleted *GlnLux* culture (pre-depletion OD_595_ = 0.025) was added to each well. Plates were sealed with non-breathable sterile film (#361006008, Fisher Scientific) to prevent media evaporation, and centrifuged for 5 s at 2000 × *g* to mix the *GlnLux* bacteria with plant extracts and M9 minimal medium. The plates were incubated at 37°C without shaking. For lux quantification, plates were read in a MicoLumatPlus LB96V luminometer with WinGlow Software (Berthold Technologies, Germany). Samples were read after 2 h of incubation for 1 s in a luminometer chamber temperature of 37°C in an endpoint assay using the integrate software function. Readings were taken every hour after incubation until lux values saturated. The plates were transferred back and forth from a 37°C incubator, and non-breathable films were replaced following each read. The 0 μg/ml Gln standard reading was subtracted from all lux values.

To test the biosensor response to exogenous Gln, six different concentrations of Gln standards (L-Gln G229, PhytoTech, 0, 625 × 10^-9^, 125 × 10^-8^, 25 × 10^-7^, 5 × 10^-6^, 1 × 10^-5^ M) were also tested in the same plates along with plant samples, following the same method (**Supplementary Figure S1**).

### Statistical Analyses

The effect of different rhizobia strains was analyzed using analysis of variance (ANOVA) set at *p* < 0.05. Means were compared using Tukey. To test for the linearity of the biosensor response, linear regression analysis was performed using a Goodness of Fit (*R*^2^) test. Total shoot N%, amount of shoot N fixed, and %Ndfa versus *GlnLux* correlation analysis were performed using the Pearson correlation test. All statistical analyses were performed using GraphPad Prism Software (v5, GraphPad Software, United States).

## Results

### Leaf Punch Screening of Inoculants Applied to Lentil

**FIGURE 2:**
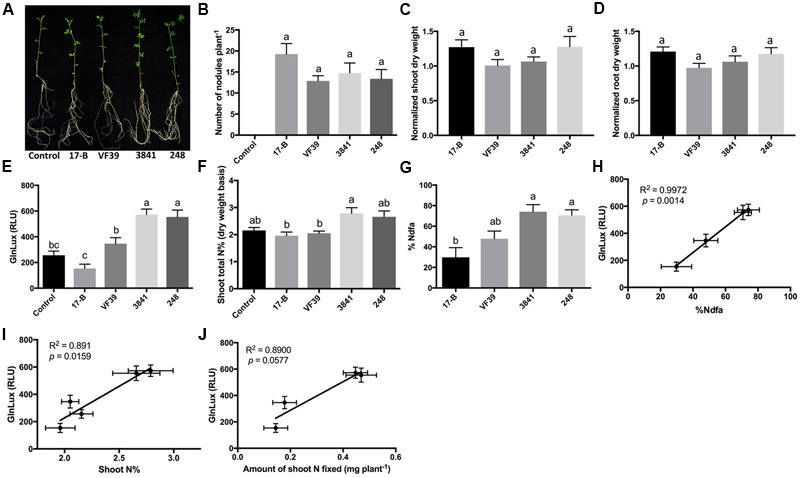
The *GlnLux* leaf punch assay to measure SNF in lentil. **(A–D)** Effect of rhizobia inoculants on morphological traits. **(A)** Representative pictures of lentil plants inoculated with four different strains of *R. leguminosarum* bv. *viciae* (wild types: 3841, VF39, 248; mutant 17-B) and the non-inoculated control. **(B)** Number of nodules per plant. The non-inoculated control had no nodules. **(C)** Normalized shoot dry matter per plant. Normalization of plant dry matter (rhizobia inoculated) was achieved by dividing dry matter of the non-inoculated plants. **(D)** Normalized root dry matter per plant. **(E–J)** Validation of the *GlnLux* luminometer assay as a method to measure SNF in an amide exporting legume (lentil) by comparing results to total N% and ^15^N dilution methods. **(E)** Corresponding *GlnLux* leaf punch outputs. A 0 μg/ml Gln standard reading was subtracted from all lux values, which were read in randomized replicates. RLU, relative lux units. **(F)** Shoot total N% analyzed using a mass spectrometer. **(G)** Percentage of N derived from the atmosphere (%Ndfa) calculated using the isotope dilution technique (see section Materials and Methods). **(H)** Correlation between *GlnLux* and %Ndfa (Pearson *R*^2^). **(I)** Correlation between *GlnLux* and shoot total N%. **(J)** Correlation between *GlnLux* and amount of shoot N fixed. For all graphs, the error bars represent the standard error of the mean (SEM) (*N* = 8). The different letters on top of each histogram indicate significant differences in the mean between treatments.

To test the *GlnLux* leaf punch assay with lentil, four strains of *R. leguminosarum* bv. *viciae* (VF39, 248, 3841, and 17-B), which differ in their N-fixation capacities, were inoculated onto a variety of lentil grown by smallholder Nepalese hillside farmers (**Figure 2A**). The different rhizobia strains all produced a similar number of root nodules (**Figure 2B** and **Supplementary Figure S2A**). A single punch per plant from a young leaf (from low-N grown lentils) was ground in PIC, co-incubated for 2 h with *GlnLux* cells in 96-well plates, and then the lux signal quantified after a 2-min read (**Figure 1B**). The *GlnLux* output (**Figure 2E**) showed a similar trend as the shoot N% (**Figure 2F**), and the percentage N derived from the atmosphere (%Ndfa) (**Figure 2G**). *GlnLux* output strongly correlated with SNF measured using the ^15^N stable isotope assay (%Ndfa) (Pearson correlation coefficient *R*^2^ = 0.9972) (**Figure 2H**), plant shoot N% (*R*^2^ = 0.8910) (**Figure 2I**), and amount of shoot N fixed (*R*^2^ = 0.890) (**Figure 2J**). The *GlnLux* result was not an artifact of altered Gln demand, as significant differences were not observed among the different rhizobia treatments for normalized shoot dry weight (**Figure 2C**), normalized root dry weight (**Figure 2D**), normalized total dry weight, shoot/root biomass ratio, root length, root surface area, or volume (**Supplementary Figures S2B–F**).

### Leaf Punch Screening of Inoculants Applied to Cowpea

**FIGURE 3:**
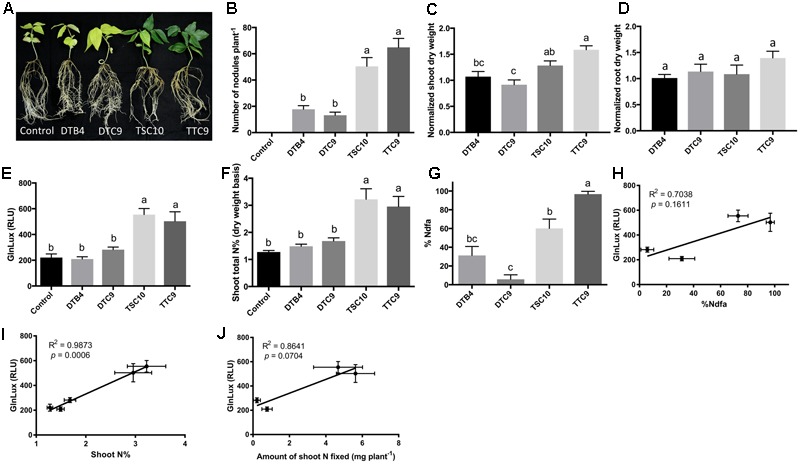
The *GlnLux* leaf punch assay to measure SNF in cowpea. **(A–D)** Effect of rhizobia inoculants on morphological traits. **(A)** Representative pictures of cowpea plants inoculated with two strains of *B. yuanmingense* (TTC9 and TSC10), one strain of *B. japonicum* (DTB4), one strain of *B. elkanii* (DTC9), and the non-inoculated control. **(B)** Number of nodules per plant. The non-inoculated control had no nodules. **(C)** Normalized shoot dry matter per plant. Normalization of plant dry matter (rhizobia inoculated) was achieved by dividing dry matter of the non-inoculated plants. **(D)** Normalized root dry matter per plant. **(E–J)** Validation of the *GlnLux* luminometer assay as a method to measure SNF in a ureide exporting legume (cowpea) by comparing results to total N% and ^15^N dilution methods. **(E)** Corresponding *GlnLux* leaf punch outputs. A 0 μg/ml Gln standard reading was subtracted from all lux values, which were read in randomized replicates. RLU, relative lux units. **(F)** Shoot total N% analyzed using a mass spectrometer. **(G)** Percentage of N derived from the atmosphere (%Ndfa) calculated using the isotope dilution technique (see section Materials and Methods). **(H)** Correlation between *GlnLux* and %Ndfa (Pearson *R*^2^). **(I)** Correlation between *GlnLux* and shoot total N%. **(J)** Correlation between *GlnLux* and amount of shoot N fixed. For all graphs, the error bars represent the SEM (*N* = 8). The different letters on top of each histogram indicate significant differences in the mean between treatments.

To test the *GlnLux* leaf punch assay with cowpea, a ureide exporter, two strains of *B. yuanmingense* (TTC9 and TSC10), one strain of *B. japonicum* (DTB4) and one strain of *B. elkanii* (DTC9), which differ in their nodulation capacity, were inoculated onto a variety of cowpea grown by smallholder Nepalese hillside farmers (**Figure 3A**). The different rhizobia strains produced a significantly different number of root nodules (*P* < 0.0001) (**Figure 3B** and **Supplementary Figure S3A**). The *GlnLux* output showed a similar trend (**Figure 3E**) as the shoot N% (**Figure 3F**) and the %Ndfa (**Figure 3G**). *GlnLux* output correlated with SNF measured using the ^15^N stable isotope assay (%Ndfa) (Pearson correlation coefficient *R*^2^ = 0.7038) (**Figure 3H**), plant shoot N% (*R*^2^ = 0.9873) (**Figure 3I**), and amount of shoot N fixed (*R*^2^ = 0.8641) (**Figure 3J**). Significant differences were not observed among the different rhizobia treatments for normalized root dry weight (**Figure 3D**), root length, root surface area, or volume (**Supplementary Figures S3B–D**). Plants inoculated with strain TTC9 had the highest shoot (**Figure 3C**) and total biomass (**Supplementary Figure S3E**), whereas the lowest biomass and shoot/root ratio were associated with strain DTC9 (**Supplementary Figures S3E,F**).

### Leaf Punch Screening of Inoculants Applied to Soybean

**FIGURE 4:**
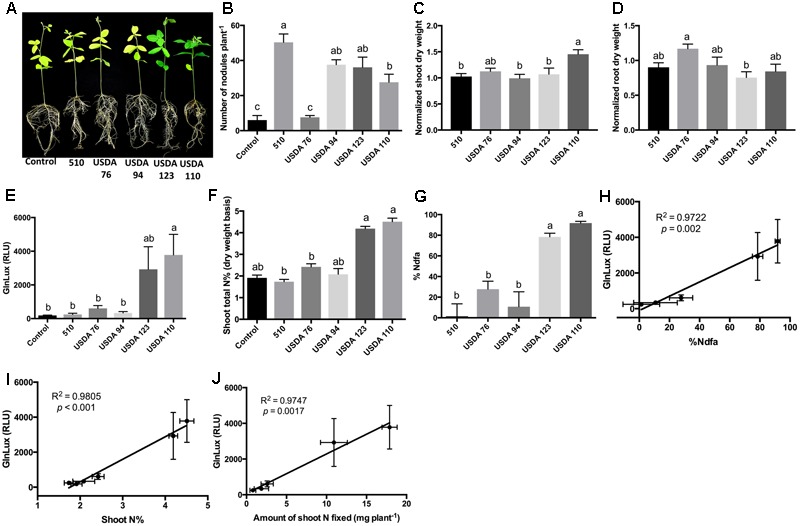
The *GlnLux* leaf punch assay to measure SNF in soybean. **(A–D)** Effect of rhizobia inoculants on morphological traits. **(A)** Representative pictures of soybean plants inoculated with three strains of *B. japonicum* (USDA 110, USDA 123, 510) and two strains of *B. elkanii* (USDA 94, USDA 76) and the non-inoculated control. **(B)** Number of nodules per plant. **(C)** Normalized shoot dry matter per plant. Normalization of plant dry matter (rhizobia inoculated) was achieved by dividing dry matter of the non-inoculated plants. **(D)** Normalized root dry matter per plant. **(E–J)** Validation of the *GlnLux* luminometer assay as a method to measure SNF in a ureide exporting legume (soybean) by comparing results to total N% and ^15^N dilution methods. **(E)** Corresponding *GlnLux* leaf punch outputs. A 0 μg/ml Gln standard reading was subtracted from all lux values, which were read in randomized replicates. RLU, relative lux units. **(F)** Shoot total N% analyzed using a mass spectrometer. **(G)** Percentage of N derived from the atmosphere (%Ndfa) calculated using the isotope dilution technique (see section Materials and Methods). **(H)** Correlation between *GlnLux* and %Ndfa (Pearson *R*^2^). **(I)** Correlation between *GlnLux* and shoot total N%. **(J)** Correlation between *GlnLux* and amount of shoot N fixed. For all graphs, the error bars represent the SEM (*N* = 8). The different letters on top of each histogram indicate significant differences in the mean between treatments.

To test the *GlnLux* leaf punch assay with soybean, another ureide exporter, a Nepalese subsistence farmer variety of soybean was inoculated with three strains of *B. japonicum* (USDA 110, USDA 123, 510) and two strains of *B. elkanii* (USDA 94 and USDA 76), which differ in their N-fixation capacities (**Figure 4A**). The different rhizobia strains produced a significantly different number of root nodules (*P* < 0.0001) (**Figure 4B** and **Supplementary Figure S4A**). The *GlnLux* output showed a similar trend (**Figure 4E**) as the shoot N% (**Figure 4F**), and the %Ndfa (**Figure 4G**). *GlnLux* output strongly correlated with SNF measured using the ^15^N stable isotope assay (%Ndfa) (Pearson correlation coefficient *R*^2^ = 0.9772) (**Figure 4H**), plant shoot N% (*R*^2^ = 0.9805) (**Figure 4I**), and amount of shoot N fixed (*R*^2^ = 0.9747) (**Figure 4J**). Significant differences were not observed among the different rhizobia treatments for root length, root surface area, or volume (**Supplementary Figures S4B–D**). However, slight differences were found among the rhizobia strains for normalized shoot (**Figure 4C**), root (**Figure 4D**), and total dry weight (**Supplementary Figure S4E**). Plants inoculated with USDA 110 (high fixer) had the highest shoot biomass, total biomass, and shoot/root ratio (**Supplementary Figure S4F**).

## Discussion

During the 1980s, the close relationship between xylem ureide composition and SNF activity was used to develop the well-known ureide assay, a technique to measure SNF in ureide exporting legumes (Herridge, 1982; Herridge and Peoples, 1990). ^15^N-methods have been used to validate the ureide method for SNF measurement (Herridge and Peoples, 1990). Similarly, here we have developed a new leaf punch-based technique to screen rhizobia inoculants for SNF activity following inoculation of both amide exporting (lentil) and ureide exporting (cowpea, soybean) legumes. The assay is based on measuring Gln output using the *GlnLux* biosensor, with minimal external N under controlled conditions. SNF-output as inferred from the *GlnLux* assay strongly correlated with the proportion of N derived from SNF measured using the ^15^N dilution method in both lentil (**Figures 2H,J**) and soybean (**Figures 4H,J**); the correlation for cowpea was lower but still strong (*R*^2^ = 0.7038) (**Figures 3H,J**). The cowpea result may have been due to the four selected rhizobia strains which represented extremes in SNF activity, either high (TSC-10 and TTC-9) or low (DTB-4 and DTC-9), with no intermediate strain(s) (**Figure 3B**). The SNF capacity of a legume can vary due to the nodule number and/or N fixation activity of a particular rhizobia strain – host genotype combination (Thilakarathna and Raizada, 2017). In lentil, differences in SNF output by the rhizobia were caused by differential SNF activity rather than nodulation (**Figures 2B,G**). In cowpea, SNF likely varied due to the dissimilar number of nodules observed between inoculants (**Figures 3B,G**), but we cannot rule out differences in SNF activity. In soybean, differences in SNF output were caused by differences in rhizobia activity and/or nodulation ability, as some inoculants (e.g., USDA 94) had high nodulation but apparent low fixation (**Figures 4B,G**).

We have compared the efficacy of the *GlnLux* method against the currently available methods to quantify SNF (**Table 1**). The *GlnLux* leaf punch test could be performed on juvenile plants, and bypassed the need for ^15^N or tissue-N analyses under the conditions used. As the biosensor and resulting assay are highly sensitive (<1 nM Gln), measurements of Gln concentrations require only leaf punches (Tessaro et al., 2012; Goron and Raizada, 2016), and in fact, in this study, single leaf punch extracts were diluted 1/100th prior to the assay. The leaf punch test is thus relatively non-destructive, and furthermore the space requirement to store samples is minimal. Hundreds of leaf punches can be assayed in a single day by one individual with minimal training, especially with automated bead-based grinding of leaf tissue. In our experience, leaf punches can be stored in -80°C for more than 1 year for later *GlnLux* analysis. All reagent costs are minimal, resulting in an assay cost of only ∼$1 USD per sample, though there is an upfront major equipment cost (for the luminometer). Finally, the assay does not require a non-N-fixing reference plant.

**Table 1:** Characteristics of methods to measure symbiotic nitrogen fixation (SNF) output (adapted from Howieson and Dilworth, 2016).

Characteristics	Technique						
	*N* balance	*N* difference	Ureide	C_2_H_2_ reduction assay	^15^N natural abundance	^15^N dilution	*GlnLux*
Laboratory			✓	✓	✓	✓	✓
Growth room/greenhouse	✓	✓	✓	✓	✓	✓	✓
Field	✓	✓	✓		✓	✓	
Non-destructive							✓
Need for a non-fixing reference plant		✓			✓	✓	
Time integrated	✓	✓			✓	✓	
Direct %Ndfa			✓		✓	✓	
Precision	Low	Low–medium	High	Low	Medium–high	Medium–high	Medium–high
High-throughput							✓
Cost	$$	$$	$$	$$	$$$	$$$	$
							
References	Rennie, 1984	Hardarson et al., 1984	Herridge, 1982	Stewart et al., 1967	Shearer and Kohl, 1986	Chalk, 1985	

*The *GlnLux* method is high-throughput, low cost, non-destructive, and there is no need for a reference plant. The *GlnLux* method can be used under controlled environmental conditions (laboratory/growth room/greenhouse) to measure SNF output with medium-high precision. However, SNF output measured by the *GlnLux* method is indirect, not time integrated, and may not precisely measure SNF output under field conditions. Symbol “✓” denotes that the specific method has the characteristic. Symbol “$” denotes the associated relative cost of the assay. The N balance method calculates SNF by considering all the N inputs and outputs on an area basis (kg N ha^-*1*^). The N difference method calculates SNF based on the difference in shoot N accumulated by N_*2*_-fixing plants vs. neighboring non-N_*2*_-fixing plants, either a single plant or on an area basis. The ureide method is based on the % ureide N in the xylem sap or stem segments. The acetylene reduction assay is based on the activity of nitrogenase enzyme, where the reduction of acetylene to ethylene by nitrogenase provides an estimate of N_2_ fixation activity. The ^*15*^N natural abundance method uses the natural difference in ^*15*^N abundance between atmospheric N and soil N to measure SNF, whereas the ^*15*^N dilution technique is based on the application of ^*15*^N-enriched N compounds to the soil. The ^15^N and ureide methods provide the percentage of N that is derived from SNF (%Ndfa). The amount of shoot N derived from SNF is calculated based on the shoot N content and %Ndfa (shoot N content × %Ndfa/100), on either a plant basis (mg N plant^-*1*^) or area basis (kg N ha^-*1*^).*

### Fixed Nitrogen Assimilation and Translocation in Legumes

Nitrogen fixed by rhizobia inside legume nodules is secreted as ammonium into the host plant cell, where it rapidly assimilated into Gln (Atkins, 1987). Gln is further metabolized into asparagine (Asn) in amide exporting legumes or as the ureide compounds, allantoin and allantoic acid, in ureide exporting legumes. Amide exporting legumes then transfer the majority of their fixed N through xylem as Asn (80%) and Gln (10%), whereas in ureide exporting legumes, the major export compounds are allantoin and allantoic acid (90%) (Schubert, 1986; Unkovich et al., 2008; Aranjuelo et al., 2011; Howieson and Dilworth, 2016). In ureide exporting legumes, aside from the ureides, Gln and Asn are the major amino acids found in xylem sap, as well as minor proportions of aspartic acid, lysine, valine, leucine, and glutamic acid (Pate et al., 1980). Indeed, xylem Gln as an immediate product of N fixation was shown to be a useful indicator of SNF activity across various legumes (Amarante et al., 2006; Amarante and Sodek, 2006; Justino and Sodek, 2013). Xylem sap can be obtained either from the stump of intact roots following decapitation of the shoot (root bleeding sap), or from freshly harvested shoots by applying a mild vacuum (vacuum-extracted sap) (Unkovich et al., 2008). In the former method, root xylem is sampled, whereas in the latter, shoot xylem is sampled. Different amide exporting legumes were shown to transfer around 10% of fixed N as Gln in the absence of external N (i.e., when SNF dependent), and low genotypic variability was shown to exist for the concentration of Gln in xylem sap across legumes (e.g., comparing *Pisum sativum, Lupinus albus*, and *Crotalaria juncea*) (Amarante et al., 2006). Similarly, low genotypic variability was found for the Asn:Gln ratio among different ureide exporting legumes (*Vigna angularis, G. max, V. unguiculata, Cyamopsis tetragonoloba, Macrotyloma uniflorum, Psophocarpus tetragonolobus, Vigna umbellata, Vigna mungo*) when plants were grown without external N (Pate et al., 1980). Nevertheless, there is variation in the type and amount of organic N exported from nodules and transferred through xylem in different legumes (Udvardi and Poole, 2013). Furthermore, complex amino acid cycling takes place in legumes in order to maintain SNF while allowing the host plant to regulate the symbiotic process (Lodwig et al., 2003). Interestingly, SNF output measured using the *GlnLux* biosensor method was highly correlated to relative Gln output in both the amide and ureide exporting legumes tested. Therefore, the *GlnLux* results suggest that Gln can be a useful indicator of symbiotic N fixation across various legumes under controlled conditions.

### Various Factors Can Affect the Glutamine Concentration in Legumes

In general, the N transport form in xylem sap changes based on soil and environmental factors (rainfall/water availability, soil nutrient availability, salinity) (Neo and Layzell, 1997; Cramer et al., 2002; Amarante et al., 2006; Bai et al., 2007; Ladrera et al., 2007; Gil-Quintana et al., 2012, 2013). The experiments in this study were undertaken under optimal, controlled conditions with minimal external N. It is important to note that external N (e.g., soil N) is also assimilated into Gln, which has the potential to confound interpretations of *GlnLux* data with respect to SNF. For example, in soybean, a ureide exporter, exposure to NH_3_ was shown to result in increased phloem Gln (Neo and Layzell, 1997) whereas another study showed similar xylem Gln levels when plants were supplied with NO_3_ (20 mM KNO_3_) compared to non-fertilized plants (McClure et al., 1979). A higher Asn:Gln ratio was observed in eight ureide exporters exposed to NO_3_, shown to be as a result of decreased Gln relative to Asn in cowpea (Pate et al., 1980). Xylem sap Gln was shown to change less significantly in amide exporting legumes in response to the source of N (SNF-dependent or external N) compared to ureide exporting legumes (Atkins et al., 1979; Amarante et al., 2006), likely because there is a larger relative background pool of Gln in the former.

In addition to external N, drought stress reduces SNF in legumes (Zahran, 1999; Giller, 2001; Kunert et al., 2016). Generally ureide exporting legumes (soybean, common bean) were shown to be more sensitive to drought stress compared to amide exporters (lentil, lupin) (Arrese-Igor et al., 2011). Therefore, the *GlnLux* assay may work more reliably with amide exporters than ureide exporters under drought stress conditions. Drought stress was shown to change Gln concentrations in amide exporters (Gil-Quintana et al., 2012) and ureide exporters (Silvente et al., 2012). The alteration in Gln concentration under drought stress varied by plant tissue (leaf/stem/root/nodule) (Ladrera et al., 2007; Gil-Quintana et al., 2012, 2013). It was found that under drought stress, leaf Gln content either decreased or remained close to the control (well-watered plants) in *Medicago truncatula* (amide exporter) (Gil-Quintana et al., 2012) and soybean (Silvente et al., 2012). Reallocation of leaf N compounds in legumes was shown to occur under drought conditions due to up-regulation of Gln synthetase (Aranjuelo et al., 2011). Further accumulation of total amino acids (Gil-Quintana et al., 2012) and proline during drought stress is a common scenario, where proline has been shown to act as an osmoregulant (Aranjuelo et al., 2011).

In addition, stress conditions that impair SNF can change the Gln concentration in xylem sap. For example, xylem Gln concentrations were shown to be reduced under water logging conditions in both amide and ureide exporting legumes (Amarante et al., 2006; Amarante and Sodek, 2006; Justino and Sodek, 2013). Interestingly, a similar pattern of reduction in xylem Gln was observed in both amide exporting (*P. sativum, L. albus, C. juncea*) and ureide exporting legumes (*G. max, Phaseolus vulgaris, V. unguiculata*) under water logging conditions similar to the reduction in SNF activity (Amarante et al., 2006). Therefore, in this study, Gln measurements were shown to be a good indicator of reductions in SNF under waterlogging stress.

Finally, Gln concentration can vary during the growth stage of legume plants. For example, Pate et al. (1980) found that the Asn:Gln ratio increased as cowpea and mung bean plants matured. However, the study did not show whether changes in the Asn:Gln ratio were due to decreased Gln or increased Asn.

### Limitations and Future Applications

The *GlnLux* leaf punch assay does not provide a direct measurement of SNF (%Ndfa) compared to ^15^N-based methods (**Table 1**). Another disadvantage is that *GlnLux* reports only the relative amount of Gln, not absolute amount. However, a concentration gradient of pure Gln standards can be used along with the leaf punch assay (**Supplementary Figure S1**), which was highly replicable. Although we found very strong correlation between *GlnLux* and ^15^N based %Ndfa, the *GlnLux* method is a temporal snapshot and does not provide an integrated measurement of accumulated SNF output over time (**Table 1**). When collecting samples, leaf punches have to be flash-frozen in liquid N and kept frozen until processing, which limits its applicability to field conditions. Finally, as noted above, stress conditions such as drought can alter the leaf Gln concentration in legumes, which challenges the use of *GlnLux* to infer SNF under unpredictable conditions. Given this caveat, and since the leaf punch method cannot distinguish between N derived from SNF versus soil (**Table 1**), it may be most suited for pre-screening rhizobia inoculants under controlled N supply conditions prior to field experiments. We also expect that the *GlnLux* leaf punch method will assist with basic research especially to understand the unsolved mechanisms involved in the maintenance of SNF by facilitating high-throughput bacteria/plant mutant screens.

## Author Contributions

Both MT and MR conceived of the manuscript. MT and MR designed the study and MT and NM conducted all experiments. MT wrote the manuscript and MR edited the manuscript. All authors discussed the results and commented on the manuscript. All authors read and approved the final manuscript.

## Conflict of Interest Statement

The authors declare that the research was conducted in the absence of any commercial or financial relationships that could be construed as a potential conflict of interest.

## Supplementary Material

FIGURE S1 Luminescence measurement of Gln standards using the *GlnLux* 96-well luminometer bioassay to demonstrate the linearity of the assay. Luminescence was measured using a concentration gradient of pure Gln standards (0, 125 × 10^-8^, 25 × 10^-7^, 5 × 10^-6^, and 1 × 10^-5^ M) using the *GlnLux* bioassay. A 0 μg/ml Gln standard reading was subtracted from all lux values, which were read in randomized replicates in an endpoint assay set to the “integrate” function. RLU, relative lux units. Error bars represent the standard error of the mean (SEM) (*N* = 3).

FIGURE S2 Nodulation, root, and shoot morphological parameters of the lentil plants inoculated with different strains of rhizobia. **(A)** Lentil roots images. **(B)** Total root length (cm plant^-1^). **(C)** Total root surface area (cm^2^ plant^-1^). **(D)** Total root volume (cm^3^ plant^-1^). **(E)** Normalized total plant dry matter per plant. **(F)** Shoot/root ratio. Error bars represent the SEM (*N* = 8). The different letters on top of each histogram indicate significant differences in the mean between treatments.

FIGURE S3 Nodulation, root, and shoot morphological parameters of the cowpea plants inoculated with different strains of rhizobia. **(A)** Cowpea roots images. **(B)** Total root length (cm plant^-1^). **(C)** Total root surface area (cm^2^ plant^-1^). **(D)** Total root volume (cm^3^ plant^-1^). **(E)** Normalized total plant dry matter per plant. **(F)** Shoot/root ratio. Error bars represent the SEM (*N* = 8). The different letters on top of each histogram indicate significant differences in the mean between treatments.

FIGURE S4 Nodulation, root, and shoot morphological parameters of the soybean plants inoculated with different strains of rhizobia. **(A)** Soybean roots images. **(B)** Total root length (cm plant^-1^). **(C)** Total root surface area (cm^2^ plant^-1^). **(D)** Total root volume (cm^3^ plant^-1^). **(E)** Normalized total plant dry matter per plant. **(F)** Shoot/root ratio. Error bars represent the SEM (*N* = 8). The different letters on top of each histogram indicate significant differences in the mean between treatments.
