# Supplementary figures and images for: A Biosensor-Based Leaf Punch Assay for Glutamine Correlates to Symbiotic Nitrogen Fixation Measurements in Legumes to Permit Rapid Screening of Rhizobia Inoculants under Controlled Conditions
Source: Front Plant Sci. 2017 Oct 9;8:1714. doi: 10.3389/fpls.2017.01714 (PMC5640704; doi:10.3389/fpls.2017.01714)

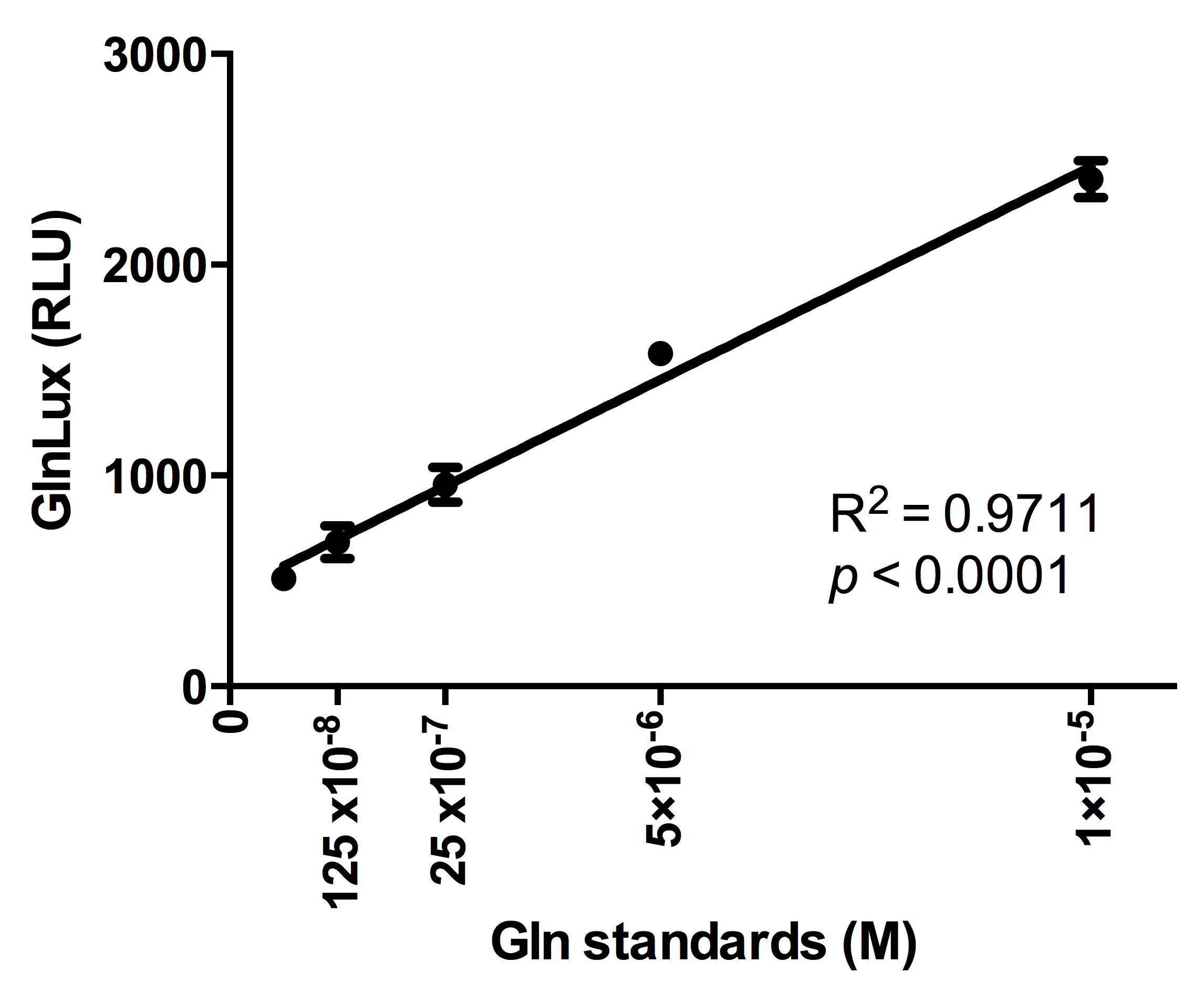

Supplement: FIGURE S1 — Luminescence measurement of Gln standards using the GlnLux 96-well luminometer bioassay to demonstrate the linearity of the assay. Luminescence was measured using a concentration gradient of pure Gln standards (0, 125 × 10-8, 25 × 10-7, 5 × 10^-6^, and 1 × 10^-5^ M) using the GlnLux bioassay. A 0 μg/ml Gln standard reading was subtracted from all lux values, which were read in randomized replicates in an endpoint assay set to the “integrate” function. RLU, relative lux units. Error bars represent the standard error of the mean (SEM) (N = 3). [file Image_1.TIFF]

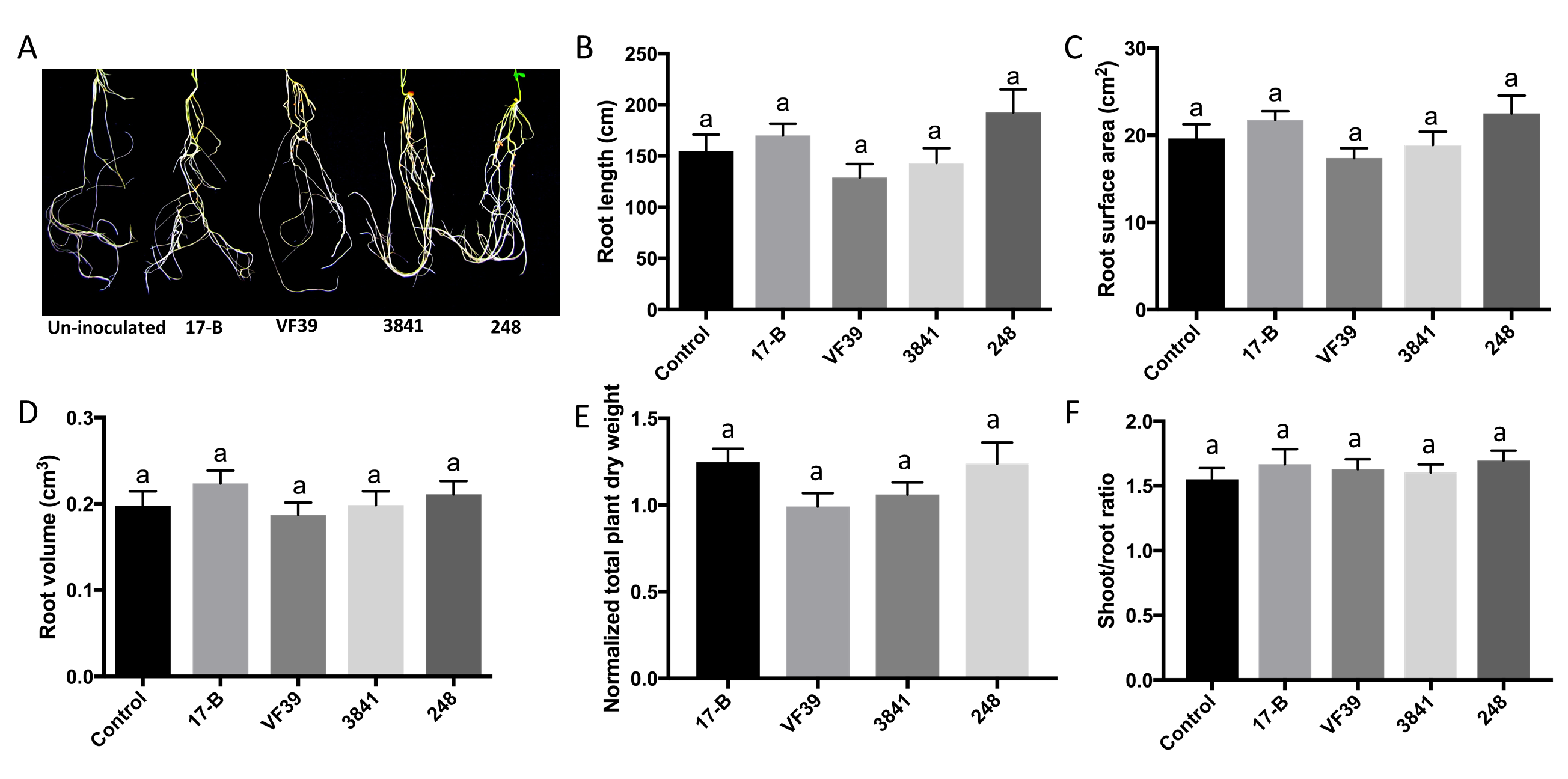

Supplement: FIGURE S2 — Nodulation, root, and shoot morphological parameters of the lentil plants inoculated with different strains of rhizobia. (A) Lentil roots images. (B) Total root length (cm plant-1). (C) Total root surface area (cm2 plant-1). (D) Total root volume (cm3 plant-1). (E) Normalized total plant dry matter per plant. (F) Shoot/root ratio. Error bars represent the SEM (N = 8). The different letters on top of each histogram indicate significant differences in the mean between treatments. [file Image_2.TIF]
